# Glances at the Hospitals

**Published:** 1900-08-11

**Authors:** 


### GLANCES AT THE HOSPITALS.

### THE NORTH STAFFORDSHIRE INFIRMARY AT HARTSHILL.

This institution was opened in 1815, and now issues its eighty-fourth annual report. The number of in-patients under treatment in 1899 was 1,977, being a decrease of 119 on that of the previous year. Of these 968 were cured, 480 relieved, not relieved 94, died 138, incurable 13, at own request 79, and 40 for various other reasons; 165 remained in the house at the end of the year. The out-patients numbered 10,812. The average residence of the in-patients was 32 days. Among the in-patients were 82 cases of enteric fever, of whom 18 died. Of 24 cases of rheumatic fever, 1 died; of 70 cases of hernia, 4 died; of 6 cases of lithotomy, all recovered; of 40 cases of cataract, 35 were successfully operated on; of 14 cases of ovariotomy, 12 were cured, 1 not relieved, and 1 died. The cost per bed occupied was £67 2s. 2d. The income from all sources was £12,148, showing a decrease of £46 on that of the previous year. The expenditure was £11,610, as against £12,104 of the previous year. Miss Jessop, formerly matron of the Grimsby Hospital, was elected lady superintendent of nurses, and Miss Cameron, formerly of the Bradford Infirmary, was elected housekeeper.

### THE LINCOLN COUNTY HOSPITAL.

This hospital was established in 1769, and since that date 107,242 patients have been treated. There were 83 patients in residence on the 31st December, 1898, and 1,265 were admitted during 1899, making a total of 1,348 under treatment. Of these 701 were discharged cured, 122 relieved, 372 were made out-patients, 29 were discharged not improved, 41 died, and 83 remain under treatment. The grand total of out-patients and in-patients was 2,888. Of the general diseases under treatment asthenia comes first with 16, acute rheumatism with 12, and chronic rheumatism with 9. All the diseases from which the patients suffered are carefully set down. Diseases of the nervous system were represented by 83 cases; of diseases of the eye there were 216, of which number 78 were senile cataract. Altogether, something like 208 distinct medical diseases were treated during the year. A great many surgical diseases were also represented, and many capital operations were performed. Chloroform was administered 430 times, ether twice, and the A.C.E. mixture once. The average duration of the treatment was four weeks and one day.

It is to be regretted that the income of the hospital has again been insufficient to meet the expenses, and the adverse balance, which in 1898 was £361, is now £403. The total income of the hospital from all sources is £25 less than in the previous year. The weekly cost of the in-patients is given at £1; but it is not stated what this sum includes. The medical staff consists of Drs. Mitchinson, Harrison, Brook, Cant, Carline and Sympson. Messrs. Woodbridge and Lock are house-surgeons, and Miss Ray is the matron.

